# Comparison of Diagnostic Values of Maternal Arginine Concentration for Different Pregnancy Complications: A Systematic Review and Meta-Analysis

**DOI:** 10.3390/biomedicines10010166

**Published:** 2022-01-13

**Authors:** Lianbin Xu, Jia Zeng, Huanan Wang, Hongyun Liu

**Affiliations:** College of Animal Sciences, Zhejiang University, Hangzhou 310058, China; lianbinxu@zju.edu.cn (L.X.); 22017088@zju.edu.cn (J.Z.)

**Keywords:** maternal arginine concentration, intrauterine growth restriction, gestational diabetes mellitus, preeclampsia, diagnostic value, pregnancy, meta-analysis

## Abstract

Abnormal arginine metabolism contributes to the development of intrauterine growth restriction (IUGR), preeclampsia (PE), and gestational diabetes mellitus (GDM), which increase the health burden of mothers and induce adverse birth outcomes. However, associations between maternal arginine concentration and different pregnancy complications have not been systematically compared. The PubMed, ScienceDirect, and Web of Science databases were searched for peer-reviewed publications to evaluate the diagnostic value of plasma arginine concentration in complicated pregnancies. Standardized mean difference (SMD) of the arginine concentration was pooled by a random effects model. The results show that increased maternal arginine concentrations were observed in IUGR (SMD: 0.48; 95% CI: 0.20, 0.76; I^2^ = 47.0%) and GDM (SMD: 0.46; 95% CI: 0.11, 0.81; I^2^ = 82.3%) cases but not in PE patients (SMD: 0.21; 95% CI: −0.04, 0.47; I^2^ = 80.3%) compared with the normal cohorts. Subgroup analyses indicated that the non-fasting circulating arginine concentration in third trimester was increased significantly in GDM and severe IUGR pregnancies, but the change mode was dependent on ethnicity. Additionally, only severe PE persons were accompanied by higher plasma arginine concentrations. These findings suggest that maternal arginine concentration is an important reference for assessing the development of pregnancy complications.

## 1. Introduction

Pregnancy complications increase the health burden of pregnant women and have negative effects on fetal development. Intrauterine growth restriction (IUGR), defined as an estimated fetal weight at or below the 10th percentile, is the second leading cause of perinatal mortality and morbidity after preterm delivery [1]. Previous works have reported that half of the stillbirths at term are neonates with a small gestational age (SGA), and most are likely to be diagnosed with IUGR [2]. Preeclampsia (PE), a serious pregnancy complication evidenced by hypertension and proteinuria after 20 weeks of gestation [3], results in elevated maternal and fetal/neonatal morbidity rates and even higher mortality [4]. In addition, the prevalence of gestational diabetes mellitus (GDM) is increasing worldwide, resulting in a higher risk of the subsequent development of type 2 diabetes mellitus (T2DM) and cardiovascular diseases in pregnant mothers [5] and health problems in newborns [6]. These findings emphasize the urgent need to improve antenatal detection and management of IUGR, PE, and GDM pregnancies.

A healthy pregnancy requires the formation of an adequately vascularized and functional placenta, and a growing body of evidence has established the relationship between placental vascular pathology and complicated pregnancies [7]. It is generally accepted that IUGR is mechanistically associated with changes in uterine and umbilical hemodynamics induced by disruptions in normal placental vasculogenesis or angiogenesis [8]. Preeclampsia is usually accompanied by the endothelial dysfunction and placental insufficiency [9]. Given that the arginine–nitric oxide (NO) pathway plays a big role in regulating vascular development and that abnormal arginine and NO availabilities lead to the vasculogenesis and angiogenesis impairments [10], maternal circulating arginine may have a strong association with the occurrence of IUGR and PE. On the other hand, there has been increasing attention paid to the role of arginine in promoting insulin secretion and improving insulin sensitivity [11,12]. A recent systematic screening of biomarkers for GDM according to the targeted metabolomic profiling revealed that an arginine-based model is most promising for predicting diabetic status [13]. The above findings suggest that plasma arginine concentration has the potential to be a predictor or biomarker for the development of several pregnancy complications. However, knowledge of the clinical details of these associations is relatively limited.

The objective of this meta-analysis of case–control studies was to compare the maternal plasma arginine concentrations between controls and different complicated pregnancies, including IUGR, PE, and GDM, as well as to investigate the operational details associated with the diagnostic value of circulating arginine.

## 2. Materials and Methods

### 2.1. Protocol and Registration

We registered in the International Prospective Register of Systematic Reviews (PROSPERO) database (No. CRD42021279651) and conducted this systematic review and meta-analysis according to the Preferred Reporting Items for Systematic Reviews and Meta-Analyses (PRISMA) guidelines [14].

### 2.2. Search Strategy

**Figure 1:**
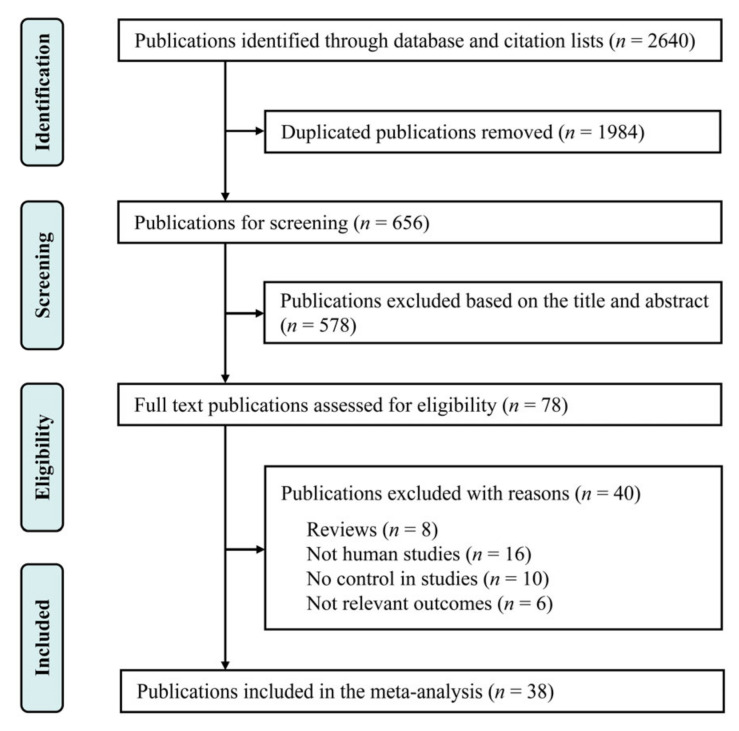
Flowchart depicting the literature search and selection strategy.

Relevant studies published by 30 September 2021 were searched in the PubMed, ScienceDirect, and Web of Science databases using the following search terms: (“intrauterine growth restriction” OR “preeclampsia” OR “gestational diabetes mellitus”) AND (“plasma arginine” OR “plasma amino acid”) AND (“pregnancy” OR “gestation period”). Our search was restricted to human studies, and no language limitations were imposed for the publications. The references cited within the selected articles were also reviewed to identify additional publications. In addition, studies published as commentaries, narrative reviews, or abstracts were excluded from this review. The selection procedure is shown in Figure 1.

### 2.3. Study Eligibility Criteria

The following criteria were taken to screen studies used for this meta-analysis: (i) the literature was a controlled clinical trial performed in humans; (ii) the study reported persons diagnosed as IUGR, PE, or GDM; (iii) the publication reported the maternal arginine concentration. Furthermore, studies that met the following exclusion criteria were removed from the meta-analysis: (i) study that was not a controlled clinical trial; (ii) study that was performed in animals; (iii) study that was conducted in patients without IUGR, PE, or GDM; (iv) the maternal arginine concentration was not included in the outcome measures; and (v) the study was a comment, case report, narrative review, or conference abstract.

### 2.4. Data Extraction

We used a standardized data collection form to extract data. Two investigators (authors L.X. and J.Z.) independently extracted information from all eligible studies for review, and the extracted data were checked again for consistency by another author (H.L.). All conflicts were resolved after discussion. Where necessary, we contacted the corresponding authors to obtain missing data for this meta-analysis. The following information was extracted from each available publication: (1) general study information, including the name of first author, title, and year of publication; (2) characteristics of participants, including location, number of persons, maternal age, plasma arginine concentration, disease severity, birth weight, and duration of pregnancy; (3) details of sampling, including sampling time and status. We extracted the risk estimates with the most adjustment.

### 2.5. Quality Assessment

The quality of all included studies was evaluated using the Newcastle–Ottawa Quality Assessment Scale (NOS), which includes selection, comparability, and outcomes [15]. The selection domain is composed of four items, comparability of one item, and outcomes of three items. A publication could receive one star for each item, receiving a maximum four stars in selection, one or two in comparability, and three in outcomes. A high risk of bias occurred when some domains did not receive stars, and in this case, the article was excluded from the analysis.

### 2.6. Statistical Analyses

Statistical analyses were conducted with Stata 14.0 software (StataCorp LP, College Station, TX, USA). The standardized mean difference (SMD) with a 95% confidence interval (CI) was calculated for the maternal arginine concentrations between patients and control groups. Pooled results for use in the forest plots were analyzed using a random effects model. Heterogeneity among the publications was estimated using the I^2^ statistic, with 0–25%, 25.1–75%, and 75.1–100% representing low, moderate, and high degrees of heterogeneity, respectively [16]. We also conducted subgroup analyses to explore the potential sources of heterogeneity, stratified by ethnicity, sampling time, sampling status, and disease severity.

Contour-enhanced funnel plots, Egger’s linear regression tests, and Begg’s rank correlation test were used to evaluate publication bias, with *p* < 0.05 as the threshold for statistical significance. Omitting one study at a time was performed to examine the effects of individual studies in sensitivity analysis [17]. Two-tailed tests were used, with *p* < 0.05 considered statistically significant.

## 3. Results

### 3.1. Study Selection and Characteristics

The study selection process and results of our literature search are shown in Figure 1. We identified 2640 studies in the PubMed, ScienceDirect, and Web of Science databases. After eliminating duplicate publications and screening titles and abstracts, 78 studies with full texts were reviewed in a detailed assessment. Among these studies, 16 were excluded because they were not performed in humans, while 16 were rejected as 10 had no controls and 6 did not report the maternal arginine concentration. Additionally, eight reviews were also removed from the meta-analysis. Finally, 38 case–control studies, including 43 clinical trials, were included in the analysis, and their characteristics are described in Table 1.

### 3.2. Maternal Arginine Concentration and IUGR

**Figure 2:**
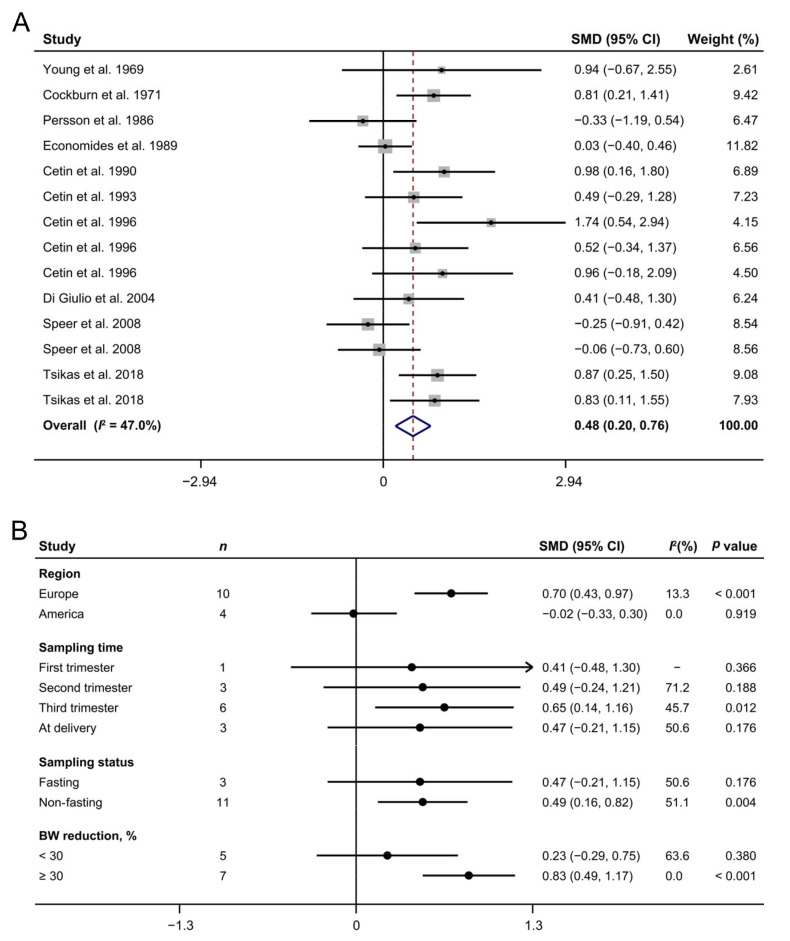
The forest plot shows the maternal arginine concentrations in intrauterine growth restriction pregnancies (**A**) as well as their subgroup analyses (**B**). The solid black circles mean the estimate of SMD. The horizontal lines indicate the 95% CI of this effect, and the sizes of the gray boxes suggest the relative weight of each publication. An open diamond denotes the overall SMD determined using a random effects model. The value on the x-axis corresponds to the SMD of maternal arginine concentration. Heterogeneity among the publications was estimated using the I^2^ statistic, with 0–25%, 25.1–75%, and 75.1–100% representing low, moderate, and high degrees of heterogeneity, respectively. BW, body weight; SMD, standard mean difference.

Ten studies [18,19,20,21,22,23,24,25,26,27] involving 153 IUGR patients and 244 controls revealed that IUGR cases had higher arginine concentrations than the normal cohorts (SMD: 0.48; 95% CI: 0.20, 0.76; I^2^ = 47.0%, Figure 2A). Subgroup analyses showed that the maternal arginine concentrations were increased in IUGR pregnant women from Europe (SMD: 0.70; 95% CI: 0.43, 0.97; I^2^ = 13.3%), but not in those from America (SMD: −0.02; 95% CI: −0.33, 0.30; I^2^ = 0%, Figure 2B). In addition, blood samples of IUGR mothers in the third trimester (SMD: 0.65; 95% CI: 0.14, 1.16; I^2^ = 45.7%) or under non-fasting status (SMD: 0.49; 95% CI: 0.16, 0.82; I^2^ = 51.1%) or whose neonatal birth weight reductions ≥30% (SMD: 0.83; 95% CI: 0.49, 1.17; I^2^ = 0%) showed higher levels of arginine compared with the control group.

### 3.3. Maternal Arginine Concentration and PE

**Figure 3:**
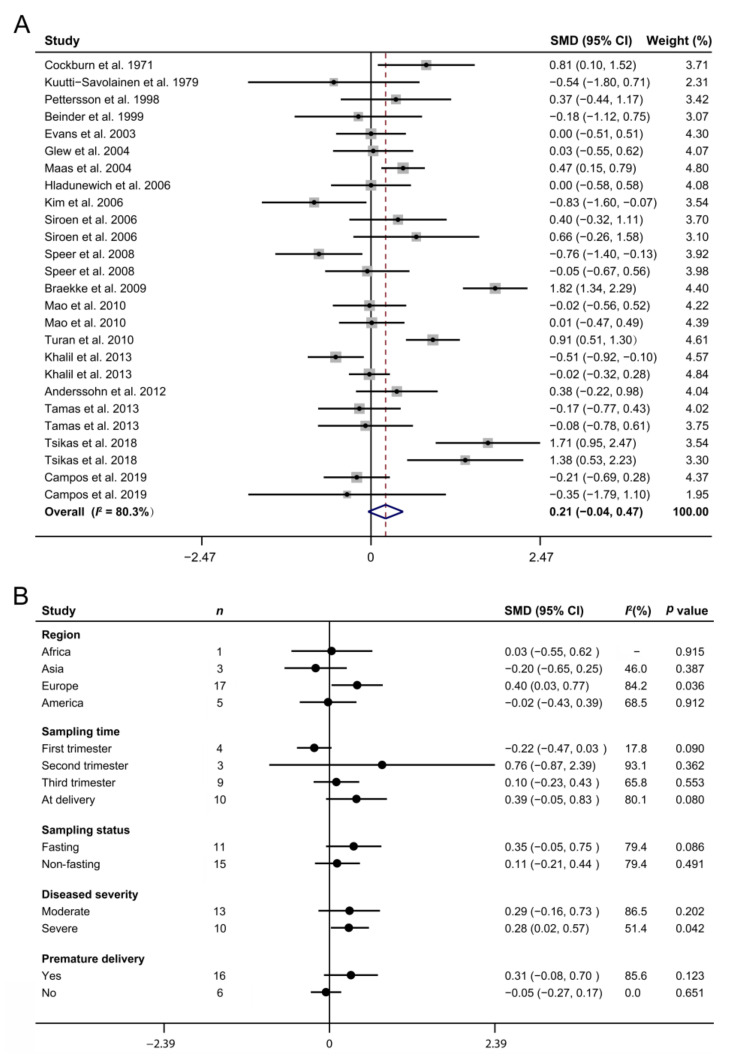
The forest plot depicts the maternal arginine concentrations in preeclampsia pregnancies (**A**) as well as their subgroup analyses (**B**). The solid black circles mean the estimate of SMD. The horizontal lines indicate the 95% CI of this effect, and the sizes of the gray boxes suggest the relative weight of each publication. An open diamond denotes the overall SMD determined using a random effects model. The value on the x-axis corresponds to the SMD of maternal arginine concentration. Heterogeneity among the publications was estimated using the I^2^ statistic, with 0–25%, 25.1–75%, and 75.1–100% representing low, moderate, and high degrees of heterogeneity, respectively. SMD, standard mean difference.

Nineteen studies [4,19,26,27,28,29,30,31,32,33,34,35,36,37,38,39,40,41,42] involving 606 cases and 849 normal pregnancies indicated that PE occurrence had no significant association with the maternal arginine concentration (SMD: 0.21; 95% CI: −0.04, 0.47; I^2^ = 80.3%, Figure 3A). However, subgroup analyses demonstrated that there were elevated circulating arginine concentrations in PE gravidas from Europe compared with the controls (SMD: 0.40; 95% CI: 0.03, 0.77; I^2^ = 84.2%, Figure 3B). Compared with normal pregnancies, the plasma arginine concentrations were increased in severe PE mothers (SMD: 0.28; 95% CI: 0.02, 0.57; I^2^ = 51.4%) but not in the moderate patients (SMD: 0.29; 95% CI: −0.16, 0.73; I^2^ = 86.5%). In addition, sampling time, sampling status, and premature delivery or not were not the causes of heterogeneity in the maternal arginine concentration (all *p* > 0.05).

### 3.4. Maternal Arginine Concentration and GDM

**Figure 4:**
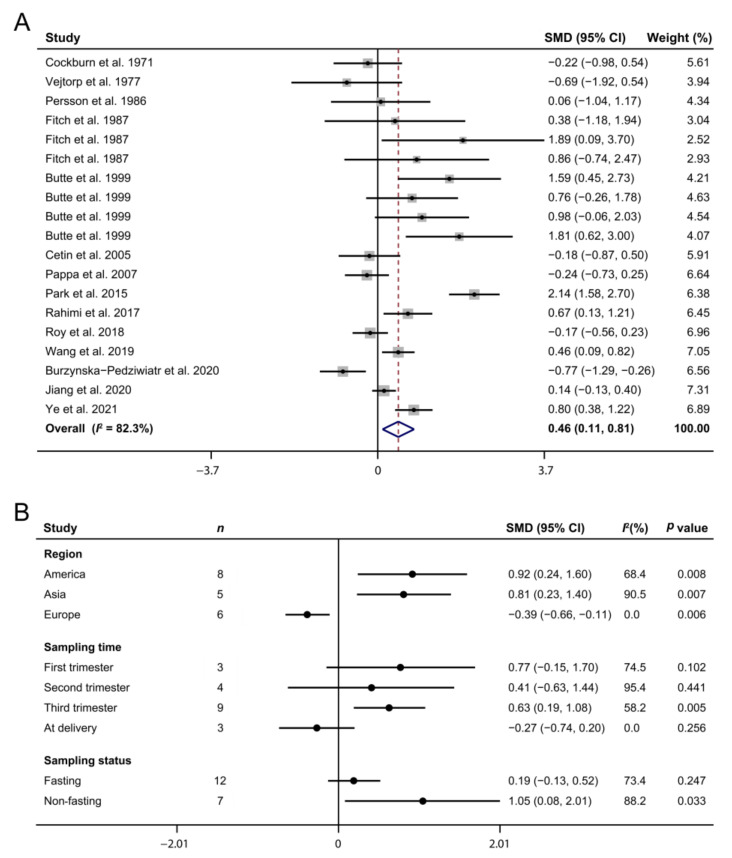
The forest plot exhibits the maternal arginine concentrations in gestational diabetes mellitus pregnancies (**A**) as well as their subgroup analyses (**B**). The solid black circles mean the estimate of SMD. The horizontal lines indicate the 95% CI of this effect, and the sizes of the gray boxes suggest the relative weight of each publication. An open diamond denotes the overall SMD determined using a random effects model. The value on the x-axis corresponds to the SMD of maternal arginine concentration. Heterogeneity among the publications was estimated using the I^2^ statistic, with 0–25%, 25.1–75%, and 75.1–100% representing low, moderate, and high degrees of heterogeneity, respectively. SMD, standard mean difference.

The association between the maternal arginine concentration and GDM was reported in 14 case–control studies [13,19,20,43,44,45,46,47,48,49,50,51,52,53], comprising 416 patients and 736 normal pregnancies. A meta-analysis indicated that the appearance of GDM was accompanied by a higher maternal arginine concentration (SMD: 0.46; 95% CI: 0.11, 0.81; I^2^ = 82.3%, Figure 4A). Subgroup analyses demonstrated that GDM mothers from America (SMD: 0.92; 95% CI: 0.24, 1.60; I^2^ = 68.4%) and Asia (SMD: 0.81; 95% CI: 0.23, 1.40; I^2^ = 90.5%) had higher arginine concentrations than those of the control group, but European mothers showed the opposite pattern (SMD: −0.39; 95% CI: −0.66, −0.11; I^2^ = 0%, Figure 4B). Furthermore, the circulating arginine concentrations in GDM pregnancies in the third trimester (SMD: 0.63; 95% CI: 0.19, 1.08; I^2^ = 58.2%) or under non-fasting status (SMD = 1.05, 95% CI: 0.08, 2.01; I^2^ = 88.2%) were higher than those in normal pregnancies.

### 3.5. Publication Bias and Sensitivity Analysis

**Figure 5:**
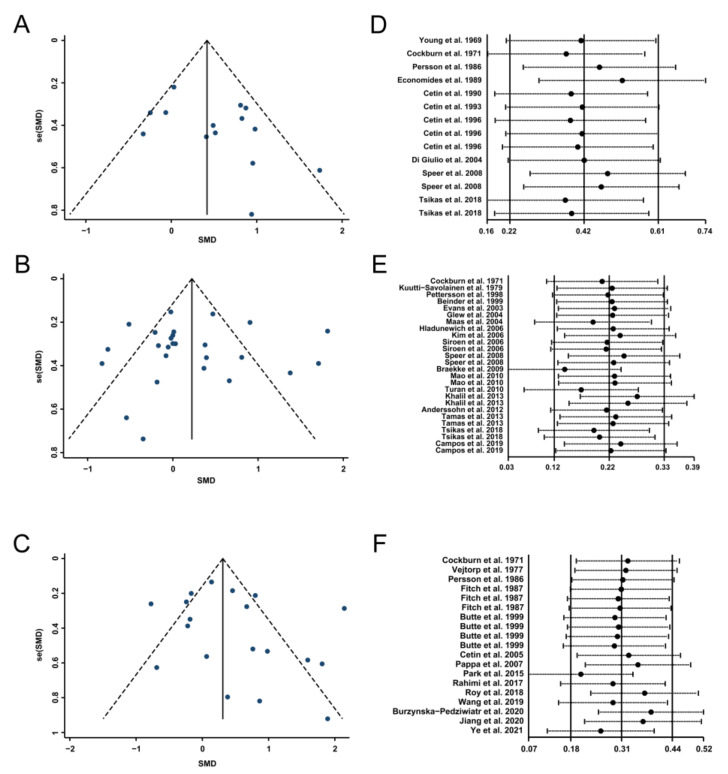
Funnel plots (**A**–**C**) and sensitivity analyses (**D**–**F**) for the included studies involved in intrauterine growth restriction, preeclampsia, and gestational diabetes mellitus, respectively. SMD, standard mean difference.

Funnel plots of the included studies for IUGR, PE, and GDM are shown in Figure 5A–C. Furthermore, Egger’s linear regression test and Begg’s rank correlation test showed no significant publication bias for each comparison (*p* > 0.05, Table 2). Sensitivity analysis revealed that no single study affected the pooled results or total effect sizes (Figure 5D–F).

## 4. Discussion

This meta-analysis analyzed data from 38 case–control studies involving a total of 1175 complicated pregnancies and 1669 healthy participants, spanning 20 countries. Our review provides the most up-to-date evidence regarding the association between maternal arginine concentration and the development of three pregnancy complications, as well as the associated interfering factors, providing important guidance for evaluating the predictive and diagnostic values of the circulating arginine concentration in IUGR, PE, and GDM.

Among the numerous metabolites, amino acids may have potential as disease biomarkers because they are involved in protein synthesis and act as metabolic regulators [54]. In particular, L-arginine, which serves as the sole endogenous precursor of NO, has a major role in the regulation of blood flow in vascular beds [55], participating in the pathogenesis of several pregnancy complications. Our study showed that the plasma arginine concentration was increased significantly in IUGR women compared with the control group, which was supported by our previous study showing that the umbilical-to-maternal ratio of the arginine concentration decreased most significantly among all essential amino acids in cases of IUGR (unpublished data). Subgroup analyses revealed that blood sampled from severe patients or in the third trimester had a more remarkable elevation in the arginine concentration, suggesting that the accumulated change in the plasma arginine level may be correlated with the severity of IUGR and that the circulating arginine concentration has the potential to act as a biomarker indicating the development of IUGR. It is worth noting that a higher arginine concentration only occurred in IUGR pregnant women from Europe but not America. One possible explanation is their diverse dietary patterns, which was confirmed by the observation that sampling status (fasting or not) is a cause of heterogeneity. The maternal plasma amino acid concentration is the balance between amino acid uptake and its utilization, including transfer to the fetus. Previous works have reported that uteroplacental uptake of amino acids from the mother may predominantly occur in the post-prandial state or may fluctuate between uptake and release, with a net transfer to the fetus over time [56]. Therefore, variation in the post-prandial maternal plasma arginine concentration may be more sensitive to an impaired placental transporter, which partly gives a practical guideline for evaluating the development of IUGR. These results indicate that the non-fasting plasma arginine concentration in the third trimester has a more effective diagnostic value for the severity of IUGR in European pregnant mothers.

Previous work established that systemic maternal endothelial cell injury and subsequent decrease in endothelium-dependent vasodilator secretion are inextricably related to hypertensive disorders in pregnancy [57], including the PE that was a new onset hypertension > 20 weeks with proteinuria. Further evidence has demonstrated that PE is causally related to markedly reduced arginine [35] and NO formation/bioavailability [58], indicating that maternal arginine concentration may have a strong association with the development of PE. On the contrary, our present study found no difference in the maternal plasma arginine concentration between normal and PE pregnancies. Despite various elements of the arginine–NO system having been widely studied in pregnant women presenting with PE, the relevant research results have not been consistent [37]. Of the included studies, 17 clinical trials found no difference in the arginine concentration between normal and PE pregnancies, while 3 trials reported that it decreased and 6 trials observed increased arginine level in PE women. We supposed that the varied PE severity among the participants in these trials, as reflected by the inclusion criteria, may partly account for the variable results, which was supported by further subgroup analyses showing that the circulating arginine concentration was only increased in severe patients compared with the control group. Previous works have demonstrated that the most important factor influencing endothelial NO production and endothelial dysfunction may be the balance between asymmetric dimethylarginine (ADMA) and arginine levels rather than the net effect of arginine concentrations [29]. Furthermore, our analysis revealed that sampling time and sampling status, as well as premature delivery or not, had no associations with the effects of PE on the plasma arginine concentration. These findings collectively indicate that the diagnostic and predictive values of the maternal arginine level alone for PE development are relatively limited, except for the patients in severe conditions and that more effective biomarkers need to be further explored. 

Previous works have reported that arginine exhibits strong insulinotropic effects and is at the nexus of several important pathways hypothesized to be involved in T2DM development [59]. Similarly, our current data show that the maternal plasma arginine concentration was significantly increased in GDM pregnancies. Despite the fact that the associations of several amino acids, such as branched-chain amino acids [60], methionine [61], and phenylalanine [45], with GDM risk have been widely described, the crucial role of arginine in the pathogenesis of GDM and its potential to be a good predictor in the earlier detection of this disease has attracted increasing attention. A recent systematic screening conducted in the GDM pregnancies with targeted metabonomics found that an arginine-based model showed the best prediction performance for diabetic status [13], which was further confirmed by a consensus multivariate analysis showing that urine arginine concentration in the first trimester has the highest accuracy in predicting GDM cases [62]. These findings have shed light on the critical role of arginine in the development of GDM. With respect to the underlying mechanism, a previous study by Piatti et al., demonstrated that arginine significantly improved insulin sensitivity in T2DM patients [63]. Hu et al., reported that arginine, which is a precursor of NO, can lead to T2DM retardation through a mechanism that includes modulating glucose homeostasis and increasing insulin sensitivity [64]. Moreover, the role of arginine in GDM may be linked to increased activity of the adenosine/arginine/nitric oxide (ALANO) pathway, which involves extracellular adenosine accumulation resulting from reduced adenosine uptake into endothelial cells [65]. Interestingly, the change mode of the circulating arginine concentration in GDM mothers from Europe was contrary to that of GDM mothers from Asia and America, suggesting that ethnicity has an important effect on the response of plasma arginine to GDM occurrence but that the underlying mechanism needs to be further investigated. Although data from non-fasting patients may show a greater differentiation of the results [50], our subgroup analyses imply that the increased plasma arginine concentration in GDM patients was more significant when sampling was taken under non-fasting status in the third trimester, providing a reference for the clinical application of the maternal arginine level in evaluating GDM development. 

In this study, we systematically reviewed the association between maternal arginine concentration and different pregnancy complications, including IUGR, PE, and GDM. On this basis, we further compared the effects of potential interfering factors, such as ethnicity, sampling time, sampling status, and disease severity, which will help us to evaluate the diagnostic value of maternal arginine in the complicated pregnancies. On the other hand, our study also has some limitations that warrant discussion. First, the studies included in our analysis were case–control designs. Therefore, publications focused on the arginine variation in early pregnancy were relatively limited, especially in the first trimester, preventing us from assessing the predictive value of arginine for pregnancy complications. Second, several studies included in this review had relatively small groups, which may partly decrease the reliability of these individual studies. Third, we may have overlooked some studies and/or missed unpublished reports, although every effort was made to contact authors in order to obtain the unpublished risk estimates.

## 5. Conclusions

Our results provide evidence that the developments of IUGR and GDM are associated with the maternal plasma arginine levels. Subgroup analyses indicate that the non-fasting circulating arginine concentrations in the third trimester were increased significantly in GDM and severe IUGR pregnant women but that the change mode was dependent on the ethnicity. Additionally, a higher arginine concentration in plasma may be an indicator for the incidence of severe PE. These findings provide practical information with diagnostic value regarding the maternal plasma arginine concentration in three pregnancy complications. Additional prospective randomized controlled trials are required to evaluate the predictive value of early pregnancy arginine concentration in the occurrence of different complicated pregnancies, specific to each individual patient’s health status.

## Figures and Tables

**Table 1:** Characteristics of the included case-control studies regarding maternal arginine concentrations in different pregnancy complications ^1^.

Reference	Country	Subjects	Sample	Age, Year	Sampling Time, wk	GA, wk	BW, kg	NOS ^2^
Young et al., 1969 [18]	Canada	Control	3		At delivery	37–40	2.90–3.50	7
		IUGR	4		At delivery	37–40	1.40–2.60	
Cockburn et al., 1971 [19]	United Kingdom	Control	26		At delivery	39.23 ± 2.61	3.30 ± 0.49	7
		IUGR	10		At delivery	36.00 ± 1.45	1.80 ± 0.23	
Persson et al., 1986 [20]	Sweden	Control	15	28.00 ± 4.67	36–39	39.29 ± 0.59	3.04 ± 0.23	7
		IUGR	8	29.20 ± 6.36	36–39	38.14 ± 0.59	2.34 ± 0.32	
Economides et al., 1989 [21]	USA	Control	62		16–36			9
		IUGR	31		16–36			
Cetin et al., 1990 [22]	Italy	Control	14		38.50 ± 0.75		3.22 ± 0.40	7
		IUGR	12		33.70 ± 3.81		1.67 ± 0.65	
Cetin et al., 1993 [23]	Italy	Control	8		26–38			8
		IUGR	31		27–38			
Cetin et al., 1996 [24]	Italy	Control	10		26–40			8
		IUGR	6		34.40 ± 1.96			
		IUGR	12		31.30 ± 3.46			
		IUGR	5		30.60 ± 2.91			
Di Giulio et al., 2004 [25]	Italy	Control	13	32.50 ± 3.97	8.50 ± 1.80	38.57 ± 3.10	3.10 ± 0.61	9
		IUGR	8	32.00 ± 1.98	8.80 ± 2.26	37.71 ± 2.43	2.12 ± 0.48	
Speer et al., 2008 [26]	USA	Control	31	21.90 ± 4.70	18.30 ± 1.60	39.50 ± 1.40	3.26 ± 0.49	9
		IUGR	12	22.90 ± 4.70	18.10 ± 1.80	39.70 ± 1.30	2.47 ± 0.22	
		Control	31	21.90 ± 4.70	At delivery	39.50 ± 1.40	3.26 ± 0.49	
		IUGR	12	22.90 ± 4.70	At delivery	39.70 ± 1.30	2.47 ± 0.22	
Tsikas et al., 2018 [27]	United Kingdom	Control	43	29.00 ± 5.30	23–25	39.40 ± 1.60	3.33 ± 0.46	8
		Control	19	27.10 ± 5.80	23–25	40.00 ± 1.70	3.21 ± 0.41	
		IUGR	14	26.30 ± 6.20	23–25	38.60 ± 4.00	2.30 ± 0.73	
Cockburn et al., 1971 [19]	United Kingdom	Control	26		At delivery	39.23 ± 2.61	3.30 ± 0.49	8
		PE	12		At delivery	36.00 ± 1.45	1.80 ± 0.23	
Kuutti-Savolainen et al., 1979 [28]	Finland	Control	7	27.00 ± 5.40	At delivery	39.60 ± 1.10	3.62 ± 0.69	6
		PE	4	23.30 ± 3.60	At delivery	40.00 ± 1.40	3.19 ± 0.26	
Pettersson et al., 1998 [29]	Sweden	Control	12	30.30 ± 3.81	32–39			7
		PE	12	29.10 ± 4.16	35.60 ± 2.77	35.60 ± 2.77	2.22 ± 0.89	
Beinder et al., 1999 [30]	Germany	Control	8	31.40 ± 3.88	At delivery	38.20 ± 2.57	3.37 ± 0.74	6
		PE	10	29.40 ± 5.25	At delivery	32.80 ± 4.88	1.95 ± 1.23	
Evans et al., 2003 [31]	USA	Control	30	24.80 ± 4.93	38.80 ± 3.29	39.50 ± 1.10	3.58 ± 0.41	8
		PE	29	27.00 ± 6.46	At delivery	35.50 ± 2.69	2.46 ± 0.82	
Glew et al., 2004 [32]	Nigeria	Control	16	26.90 ± 7.20	35.30 ± 5.50			9
		PE	37	25.00 ± 7.30	35.40 ± 3.90			
Maas et al., 2004 [33]	Colombia	Control	93	19.60 ± 3.70	At delivery	39.00 ± 3.70	3.21 ± 0.43	8
		PE	67	20.10 ± 5.40	At delivery	36.00 ± 1.63	2.46 ± 0.78	
Hladunewich et al., 2006 [34]	USA	Control	22	32.00 ± 5.00	At delivery	39.80 ± 0.90		7
		PE	23	28.00 ± 7.00	At delivery	34.40 ± 3.70		
Kim et al., 2006 [35]	Korea	Control	13	31.10 ± 3.70	>20	39.10 ± 2.20		8
		PE	16	30.70 ± 4.70	>20	35.90 ± 3.90		
Siroen et al., 2006 [36]	The Netherlands	Control	15	32.00 ± 5.00	At delivery	41.30 ± 1.10	3.52 ± 0.56	7
		PE	16	31.00 ± 5.00	At delivery	37.10 ± 2.90	2.51 ± 0.86	
		PE	7	30.00 ± 6.00	At delivery	32.40 ± 5.90	1.70 ± 1.20	
Speer et al., 2008 [26]	USA	Control	31	21.90 ± 4.70	18.30 ± 1.60	39.50 ± 1.40	3.26 ± 0.49	9
		PE	15	25.10 ± 5.80	16.90 ± 3.20	35.90 ± 3.80	2.48 ± 0.85	
		Control	31	21.90 ± 4.70	At delivery	39.50 ± 1.40	3.26 ± 0.49	
		PE	15	25.10 ± 5.80	At delivery	35.90 ± 3.80	2.48 ± 0.85	
Braekke et al., 2009 [37]	Norway	Control	51	32.50 ± 5.75	At delivery	38.70 ± 1.83	3.47 ± 0.47	7
		PE	47	31.50 ± 5.75	At delivery	32.90 ± 3.45	1.72 ± 0.75	
Mao et al., 2010 [38]	China	Control	30					7
		PE	24					
		PE	38					
Turan et al., 2010 [39]	Turkey	Control	54	29.60 ± 5.90	33.90 ± 4.20			8
		PE	55	32.00 ± 6.60	33.50 ± 4.10			
Khalil et al., 2013 [40]	United Kingdom	Control	300	32.50 ± 6.89	12.40 ± 0.89	40.00 ± 1.41	3.40 ± 0.44	9
		PE	25	30.20 ± 8.67	12.40 ± 0.44	32.00 ± 2.52	1.40 ± 0.37	
		PE	50	32.40 ± 5.93	12.40 ± 0.59	38.90 ± 1.63	3.10 ± 0.74	
Anderssohn et al., 2012 [41]	Germany	Control	28	31.70 ± 5.30	36.60 ± 3.50	39.00 ± 1.00	3.56 ± 0.43	7
		PE	18	31.90 ± 6.20	33.90 ± 3.30	35.00 ± 3.50	2.22 ± 0.95	
Tamás et al., 2013 [42]	Hungary	Control	15	30.10 ± 6.50	29.80–39.20	39.20 ± 1.30	3.41 ± 0.56	8
		PE	36	30.00 ± 6.80	29.80–39.20	31.80 ± 2.90	1.45 ± 0.54	
		PE	17	31.20 ± 6.20	29.80–39.20	38.00 ± 1.60	3.03 ± 0.60	
Tsikas et al., 2018 [27]	United Kingdom	Control	43	29.00 ± 5.30	23.00–25.00	39.40 ± 1.60	3.33 ± 0.46	8
		Control	19	27.10 ± 5.80	23.00–25.00	40.00 ± 1.70	3.21 ± 0.41	
		PE	10	28.20 ± 4.70	23.00–25.00	34.80 ± 3.50	2.07 ± 0.72	
Campos et al., 2019 [4]	Switzerland	Control	33	30.40 ± 3.93	12.40 ± 0.74	39.60 ± 1.06	3.43 ± 0.52	8
		PE	33	31.00 ± 4.81	12.40 ± 0.53	37.90 ± 2.33	3.03 ± 0.74	
		Control	3	31.90 ± 2.07	12.29 ± 1.16	40.43 ± 0.11	3.01 ± 0.81	
		PE	5	27.00 ± 5.78	12.57 ± 0.32	37.43 ± 2.12	2.82 ± 1.02	
Cockburn et al., 1971 [19]	United Kingdom	Control	26		At delivery	39.23 ± 2.61	3.30 ± 0.49	7
		GDM	9		At delivery	36.00 ± 1.15	3.80 ± 0.58	
Vejtorp et al., 1977 [43]	Denmark	Control	5	28.00 ± 2.03	At delivery	39.60 ± 1.17	3.90 ± 0.49	7
		GDM	6	25.80 ± 2.34	At delivery	37.70 ± 0.87	2.80 ± 0.33	
Persson et al., 1986 [20]	Sweden	Control	15	28.00 ± 4.67	36.00–39.00	39.29 ± 0.59	3.04 ± 0.23	7
		GDM	4	30.80 ± 5.20	36.00–39.00	39.29 ± 0.28	3.66 ± 0.49	
Fitch et al., 1987 [44]	USA	Control	8	26.80 ± 7.40	30.00–36.00		3.33 ± 0.59	7
		GDM	2	28.50 ± 7.80	30.00–36.00		3.65 ± 0.10	
Butte et al., 1999 [45]	USA	Control	8	23.00 ± 2.00	6.00	39.40 ± 1.80	3.70 ± 0.40	7
		GDM	8	28.00 ± 5.00	6.00	39.50 ± 0.80	3.60 ± 0.40	
		Control	8	23.00 ± 2.00	32–36	39.40 ± 1.80	3.70 ± 0.40	
		GDM	8	28.00 ± 5.00	32–36	39.50 ± 0.80	3.60 ± 0.40	
Cetin et al., 2005 [46]	Italy	Control	16		At delivery			9
		GDM	17		At delivery			
Pappa et al., 2007 [47]	Greece	Control	46	27.85 ± 4.99	30–33			8
		GDM	25	27.84 ± 5.14	30–33			
Park et al., 2015 [48]	Korea	Control	25	33.30 ± 3.80	26.24 ± 2.07			9
		GDM	64	33.70 ± 4.10	26.36 ± 1.87			
Rahimi et al., 2017 [49]	Iran	Control	25	29.46 ± 5.45	>25			9
		GDM	31	32.65 ± 5.56	>25			
Roy et al., 2018 [50]	Canada	Control	50	31.00 ± 3.80	24–28	38.40 ± 1.70	3.15 ± 0.75	9
		GDM	50	31.00 ± 3.70	24–28	39.30 ± 1.50	3.30 ± 0.52	
Wang et al., 2019 [51]	China	Control	63	29.32 ± 3.61	26.06 ± 1.64			7
		GDM	58	30.75 ± 4.23	26.04 ± 1.23			
Burzynska-Pedziwiatr et al., 2020 [13]	Poland	Control	35	28.00 ± 3.70	24–28			9
		GDM	29	30.00 ± 4.44	24–28			
Jiang et al., 2020 [52]	China	Control	366	28.10 ± 3.60	12–16			9
		GDM	65	29.80 ± 3.80	12–16			
Ye et al., 2021 [53]	China	Control	48	28.22 ± 3.02	39.12 ± 1.15			7
		GDM	48	28.09 ± 3.35	39.17 ± 1.18			

^1^ Data are presented as the mean ± SD or range. BW, body weight; GA, gestational age; GDM, gestational diabetes mellitus; HD, hypertension disorder; IUGR, intrauterine growth restriction. ^2^ The quality of all included studies was evaluated using the Newcastle–Ottawa Quality Assessment Scale (NOS, [15]), which includes selection, comparability, and outcomes.

**Table 2:** Publication bias examined by Egger’s linear regression test and Begg’s rank correlation test ^1^.

Pregnancy Complication	*p* for Egger’s Test	*p* for Begg’s Test
IUGR	0.109	0.511
PE	0.873	0.508
GDM	0.311	0.184

^1^ IUGR, intrauterine growth restriction; GDM, gestational diabetes mellitus; PE, preeclampsia.

## Data Availability

The data presented in this study are available on request from the corresponding author.
